# Risk assessment for mortality in patients with ST‐elevation myocardial infarction undergoing primary percutaneous coronary intervention: A retrospective cohort study

**DOI:** 10.1002/hsr2.1867

**Published:** 2024-02-13

**Authors:** Alireza Oraii, Melika Shafeghat, Haleh Ashraf, Abbas Soleimani, Sina Kazemian, Azadeh Sadatnaseri, Naser Saadat, Khashayar Danandeh, Ashley Akrami, Pargol Balali, Mohamadreza Fatahi, Shahrokh Karbalai Saleh

**Affiliations:** ^1^ Students' Scientific Research Center Tehran University of Medical Sciences Tehran Iran; ^2^ School of Medicine Tehran University of Medical Sciences Tehran Iran; ^3^ Feinberg School of Medicine Northwestern University Chicago Illinois USA; ^4^ Cardiac Primary Prevention Research Center, Cardiovascular Diseases Research Institute Tehran University of Medical Sciences Tehran Iran; ^5^ Research Development Center, Sina Hospital Tehran University of Medical Sciences Tehran Iran; ^6^ Department of Cardiology, Sina Hospital Tehran University of Medical Sciences Tehran Iran; ^7^ Chicago College of Osteopathic Medicine Midwestern University Downers Grove Illinois USA

**Keywords:** 1‐year mortality, in‐hospital mortality, major adverse cardiac events, PCI, STEMI

## Abstract

**Background and Aims:**

Primary percutaneous coronary intervention (PCI) is the treatment of choice in ST‐elevation myocardial infarction (STEMI) patients. This study aims to evaluate predictors of in‐hospital and long‐term mortality among patients with STEMI undergoing primary PCI.

**Methods:**

In this registry‐based study, we retrospectively analyzed patients with STEMI undergoing primary PCI enrolled in the primary angioplasty registry of Sina Hospital. Independent predictors of in‐hospital and long‐term mortality were determined using multivariate logistic regression and Cox regression analyses, respectively.

**Results:**

A total of 1123 consecutive patients with STEMI were entered into the study. The mean age was 59.37 ± 12.15 years old, and women constituted 17.1% of the study population. The in‐hospital mortality rate was 5.0%. Multivariate analyses revealed that older age (odds ratio [OR]: 1.06, 95% confidence interval [CI]: 1.02–1.10), lower ejection fraction (OR: 0.97, 95% CI: 0.92–0.99), lower mean arterial pressure (OR: 0.95, 95% CI: 0.93–0.98), and higher white blood cells (OR: 1.17, 95% CI: 1.06–1.29) as independent risk predictors for in‐hospital mortality. Also, 875 patients were followed for a median time of 21.8 months. Multivariate Cox regression demonstrated older age (hazard ratio [HR] = 1.04, 95% CI: 1.02–1.06), lower mean arterial pressure (HR = 0.98, 95% CI: 0.97–1.00), and higher blood urea (HR = 1.01, 95% CI: 1.00–1.02) as independent predictors of long‐term mortality.

**Conclusion:**

We found that older age and lower mean arterial pressure were significantly associated with the increased risk of in‐hospital and long‐term mortality in STEMI patients undergoing primary PCI. Our results indicate a necessity for more precise care and monitoring during hospitalization for such high‐risk patients.

## 1 INTRODUCTION

According to the current estimates of the Global Burden Disease Study (GBD) 2019, cardiovascular diseases (CVDs), with more than 390 million disability‐adjusted life years, were the primary cause of disability worldwide.^1^ Despite the public perception that CVD mortality has decreased due to the development of therapeutic equipment, it has increased dramatically from 12.1 to 18.6 million since 1990.^1^, ^2^ Furthermore, acute myocardial infarction (AMI) is the leading cause of global CVDs‐related deaths.^3^ Hence, several studies reevaluated the predisposing factors that lead AMI to fatal consequences. In this regard, recent evidence has found a greater mortality rate for ST‐elevation myocardial infarction (STEMI) compared to non‐STEMI, while multiple guidelines and health policies have been implemented to address this problem.^4^, ^5^ The latest guidelines have emphasized the benefits of a timely primary percutaneous coronary intervention (PCI) over thrombolytic therapy in patients presenting with STEMI.^6^, ^7^

Despite all the medical advances, it is noteworthy to acknowledge that STEMI remains one of the major health concerns giving rise to multiple patient transfer protocols, new generation stents, and updated pharmacotherapy.^8^, ^9^ Numerous studies, including national and regional registries, have enrolled patients with acute coronary syndrome to gather epidemiological data regarding patient characteristics, cardiovascular risk factors, treatment strategies, and outcomes.^10^, ^11^, ^12^, ^13^, ^14^ Subgroups of patients presenting with STEMI have been evaluated in these studies to find the predictors of adverse outcomes and complications.^15^, ^16^ However, these studies have assessed an unselected group of STEMI patients regarding time delay in presentation and use of thrombolytic agents, which confounds generalizing the effect of different variables on adverse cardiovascular outcomes in diverse patient populations.

This study aims to assess the characteristics of patients undergoing primary PCI after STEMI. Also, we have investigated the predictors of in‐hospital and long‐term mortality in such patients in a high‐volume referral center in Tehran, Iran.

## 2 MATERIAL AND METHODS

### 2.1 Study design

This is a retrospective cohort study derived from the Primary Angioplasty Registry of Sina Hospital (PARS) between November 2016 and September 2021. Our study adheres to the strengthening the reporting of observational studies in epidemiology (STROBE) guidelines.^17^ Methodological details of the PARS registry have been previously published.^18^ In brief, the PARS registry is an ongoing prospective hospital‐based index of patients undergoing primary PCI for STEMI in the Sina Hospital, a tertiary teaching hospital affiliated with the Tehran University of Medical Sciences located in the southern region of Tehran, Iran.

Myocardial injury is defined by a minimum of one unit rising or falling above the 99th percentile of the upper reference range of cardiac troponin. Hence, a cardiac injury within the context of AMI symptoms is defined as myocardial infarction (MI). MI with incompatibility to electrocardiographic (ECG) features is STEMI. New ST‐segment elevation was considered at the J point in two contiguous leads of ≥1 mm other than leads V_2_–V_3._ For leads V_2_–V_3,_ the following cut points apply: ≥2.5 mm in men younger than 40 years old, ≥2 mm in men older than 40 years old, or ≥1.5 mm in women regardless of age. Furthermore, AMI is indicated in newly onset or presumed new left bundle branch block (LBBB) with ST‐segment elevation of ≥1 mm concordant with the QRS complex in any lead.^19^

This registry began enrolling patients in November 2016 after implementing “Project‐247” by the Iranian Ministry of Health and Medical Education. In addition, patients who were referred from the emergency department and other nearby hospitals were included in the registry. The term “Project‐247” derived from 24‐h‐a‐day and 7‐days‐a‐week availability, which was designed to reach a faster out‐patient diagnosis of STEMI in individuals complaining of ischemic heart disease and their principal manifestations. After the diagnosis of STEMI, patients will be directly transferred to the nearest participating PCI‐capable hospital. In the meantime, a skilled team of cardiac interventionists and trained staff at the designated center is informed to be ready for coronary catheterization and possible PCI upon the arrival of the patient.

### 2.2 Study population

In this study, we included patients aged ≥18 years old with a diagnosis of acute STEMI who underwent primary PCI within 12 h of pain onset from November 2016 to September 2021. On the other hand, patients aged <18 years old as well as who experienced pain onset to reach primary PCI of more than 12 h, and those who received thrombolytic therapy before PCI were excluded from the study. In addition, patients for whom coronary angiography was not performed or other diagnoses such as Takotsubo cardiomyopathy was suggested during catheterization were also excluded.

### 2.3 Data collection

In addition to paper‐based medical records, predesignated staff members such as medical doctors and trained research nurses were all trained to document all medical records into an electronic database. This real‐time web‐based electronic database includes all demographic data, laboratory test results, echocardiographic features, angiographic findings, and coronary interventions and procedures details. Staff cardiologists noted medications, medical treatments, unfavorable outcomes, and in‐hospital complications. We have followed up 875 patients (77.9% of the whole cohort population) for a median of 21.8 months using phone calls after discharge. The other patients were unavailable and nonresponsive to phone calls after multiple tries.

Before primary PCI, all patients were given 325 mg aspirin and 600 mg clopidogrel, as well as anticoagulation. In instances with a significant thrombus load or insufficient anticoagulation, a glycoprotein IIb/IIIa inhibitor was additionally given at the discretion of the cardiologists. Patients were medically treated according to the current American College of Cardiology (ACC), the American Heart Association (AHA), and the European Society of Cardiology (ESC).^20^ Patients were carefully interviewed to investigate cardiovascular risk factors such as hypertension, diabetes, and dyslipidemia on admission. Furthermore, they were closely monitored for atrial and ventricular arrhythmias, significant bleeding episodes, requiring inotropes or devices for hemodynamic support, recurring chest discomfort, and ischemic or hemorrhagic stroke. All patients were followed up for in‐hospital mortality or all‐cause mortality after discharge.

### 2.4 Statistical analysis

Continuous variables with normal distribution were reported as means with standard deviations and were compared using independent *T* tests. The Mann–Whitney *U* test was used for the continuous variables with skewed distribution, and they were reported as median [interquartile range]. Categorical variables were presented as percentages and absolute numbers, and *χ* ^2^ and Fisher exact tests were also used to compare them. Variables with a missing rate >20% were excluded from the whole study. Afterward, we utilized univariate logistic regression analysis to determine predictors of the in‐hospital and long‐term mortalities. During the model selection process, we excluded certain significant variables due to their collinearity with others. For instance, among variables such as history of hypertension, mean systolic blood pressure (SBP), mean diastolic blood pressure (DBP), mean arterial pressure (MAP), and pulse pressure, we opted to include only MAP in the regression models. This decision was made to avoid redundancy and multicollinearity, thereby enhancing the reliability of our statistical models. In the multivariable logistic regression analysis, variables with a *p* value < 0.05 in univariate results were further assessed in a backward step‐wise analysis. In this analysis, the criteria for variable entry into the model was set at a significance level of 0.05, while the removal of variables from the model was set at a significance level of 0.10. The odds ratios (ORs) and 95% confidence intervals (CIs) were reported for variables of the mentioned analyses. To assess long‐term mortality, we used univariate and multivariate Cox regression analysis. Afterward, we addressed independent predictors of follow‐up mortality for our patients. The hazard ratio and 95% confidence interval were reported for variables of the mentioned analyses. All statistical tests were two‐sided and a *p* value < 0.05 was considered significant. Statistical analyses were performed using SPSS version 24.0 (IBM SPSS Inc).

## 3 RESULTS

**Figure 1:**
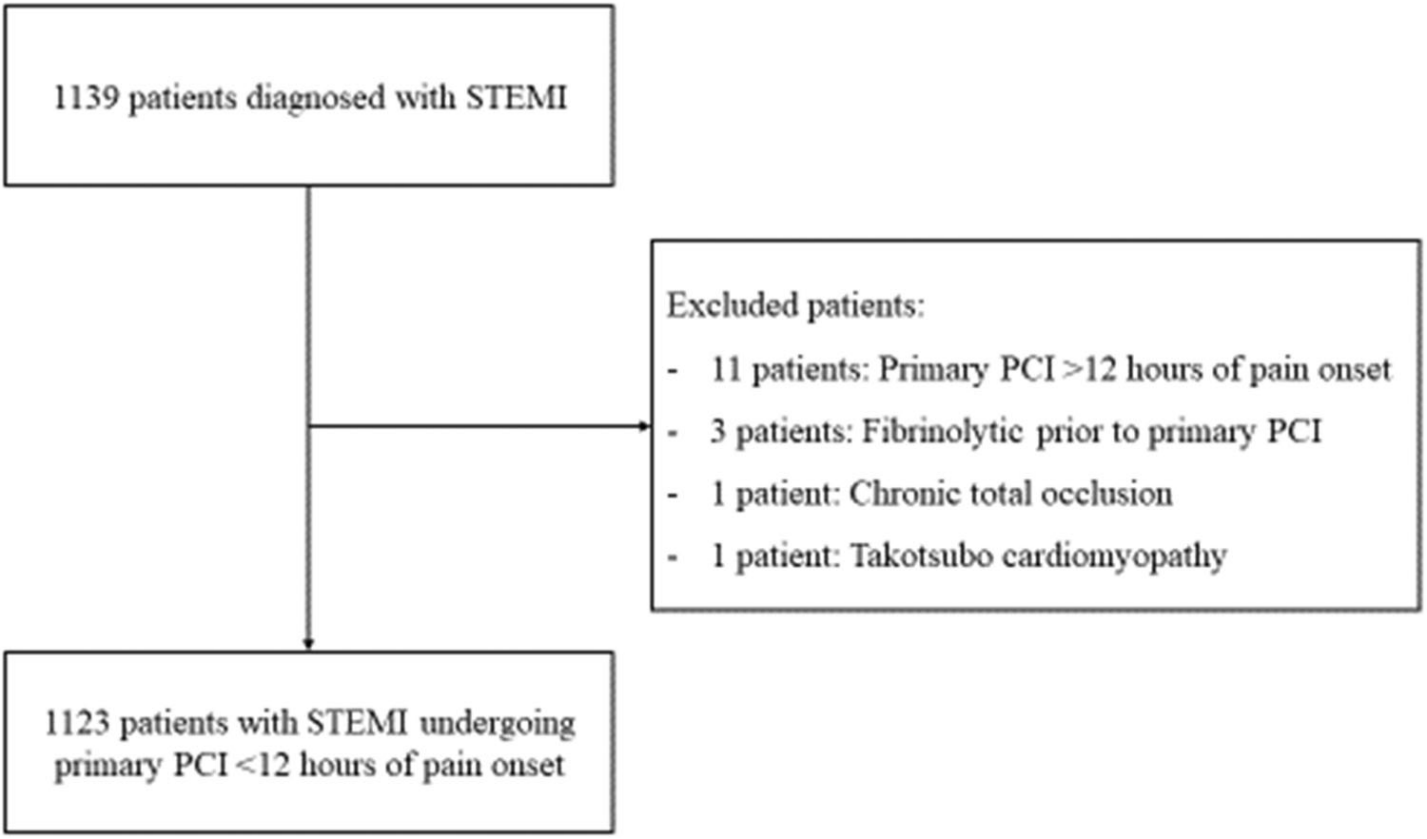
Study flow diagram. PCI, percutaneous coronary intervention; STEMI, ST‐elevation myocardial infarction.

A total of 1139 patients were diagnosed with acute STEMI from November 2016 to September 2019, all of whom underwent primary PCI. Eleven patients presented after 12 h of pain onset and three patients had previously administered thrombolytic agents at different medical centers; hence, they were excluded from the study. Furthermore, two additional patients were ruled out due to their diagnosis of chronic total occlusion and Takotsubo cardiomyopathy. Ultimately, the final study population comprised 1123 STEMI patients who underwent primary PCI within the critical 12‐h window following the onset of pain (Figure 1). In this study, in‐hospital mortality was observed in 56/1123 (5.0%) of patients, while long‐term mortality happened in 124/875 (14.2%) of cases.

### 3.1 Baseline characteristics

The baseline characteristics of the study population are presented in Table 1. The mean age of patients was 59.37 ± 12.15 years and 82.9% were male. Hypertension was found to be the most prevalent risk factor for coronary artery disease 470/1123 (41.9%), followed by smoking history 448/1123 (39.9%). Diabetes mellitus was reported in 336/1123 (29.9%) of patients and 137/1123 (12.2%) patients had previously undergone coronary artery revascularization. Factors such as older age, a history of hypertension, and prior coronary revascularization were significantly linked to higher in‐hospital mortality (Table 1). These risk factors were further investigated in relation to follow‐up mortality, revealing that older age, female sex, history of hypertension, and smoking were associated with an increased risk of long‐term mortality (Table 2). Upon initial admission and patient evaluation, lower mean SBP (104.14 ± 31.17 vs. 131.87 ± 26.14 mmHg; *p* value: 0.001) and lower mean DBP (62.94 ± 16.58 vs. 74.81 ± 10.96 mmHg; *p* value: 0.001) were significantly observed more among the deceased group. Lower left ventricular ejection fraction (LVEF), which was detected on the first day after PCI, was more likely to be observed in the deceased group (33.03 ± 15.72 vs. 43.15 ± 10.80; *p* value: 0.002). A higher number of white blood cells (14.14 ± 4.73 vs. 11.52 ± 3.58; *p* value: 0.002), increased amount of blood urea (55.96 ± 48.07 vs. 34.39 ± 15.44; *p* value: 0.007) and blood/serum creatinine (1.44 ± 0.48 vs. 1.13 ± 0.55; *p* value: 0.001) were all significantly higher in the deceased group. Among 875 patients who followed up to assess long‐term mortality, all echocardiographic variables, including lower ejection fraction (38.87 ± 13.74 vs. 43.25 ± 10.90; *p* value: 0.004), SBP (119.82 ± 33.75 vs. 131.74 ± 25.90; *p* value: 0.001), DBP (69.64 ± 15.97 vs. 75.03 ± 11.11; *p* value: 0.001), MAP (86.37 ± 21.03 vs. 93.93 ± 15.17; *p* value: <0.001), and pulse pressure (50.17 ± 21.97 vs. 56.71 ± 18.48; *p* value: 0.004) were significantly different in the two groups.

**Table 1:** Comparison of baseline, echocardiographic, and angiographic characteristics of STEMI patients undergoing primary PCI among in‐hospital mortality and discharged groups.

Characteristics^a^		Total (*N* = 1123)	In‐hospital deceased (*N* = 56)	Discharged (*N* = 1067)	*p* Value^b^
Demographics					
Age (years)		59.37 ± 12.15	67.96 ± 12.58	58.92 ± 11.96	**0.001**
Sex					
Male		931/1123 (82.9%)	42/56 (75.0%)	889/1067 (83.3%)	0.11
Female		192/1123 (17.1%)	14/56 (25.0%)	178/1067 (16.7%)	
Comorbidities					
Hypertension		470/1123 (41.9%)	16/56 (28.6%)	454/1067 (42.5%)	**0.04**
Diabetes mellitus		336/1123 (29.9%)	13/56 (23.2%)	323/1067 (30.3%)	0.26
Dyslipidemia		460/1123 (41.0%)	6/56 (10.7%)	454/1067 (42.5%)	**0.001**
Smoking		448/1123 (39.9%)	4/56 (7.1%)	444/1067 (41.6%)	**0.001**
Coronary revascularization		137/1123 (12.2%)	12/56 (21.4%)	125/1067 (11.7%)	**0.03**
Hemodynamic and echocardiographic data					
Left ventricle ejection fraction (%)		42.87 ± 11.09	33.03 ± 15.72	43.15 ± 10.80	**0.002**
Systolic blood pressure (mmHg)		130.46 ± 27.10	104.14 ± 31.17	131.87 ± 26.14	**0.001**
Diastolic blood pressure (mmHg)		74.21 ± 11.60	62.94 ± 16.58	74.81 ± 10.96	**0.001**
Mean arterial pressure (mmHg)		92.95 ± 15.95	76.67 ± 20.71	93.83 ± 15.19	**0.001**
Pulse pressure (mmHg)		56.24 ± 18.97	41.20 ± 18.74	7.05 ± 18.65	**0.001**
Laboratory data					
White blood cells (×10^9^/L)		11.61 ± 3.66	14.14 ± 4.73	11.52 ± 3.58	**0.002**
Hemoglobin (g/dL)		14.23 ± 2.00	13.71 ± 2.87	14.25 ± 1.96	0.26
Platelets (×10^9^/L)		227.18 ± 70.63	246.68 ± 88.01	226.43 ± 69.82	0.08
Urea (mg/L)		35.23 ± 18.28	55.96 ± 48.07	34.39 ± 15.44	**0.007**
Creatinine (mg/dL)		1.14 ± 0.55	1.44 ± 0.48	1.13 ± 0.55	**0.001**
Creatinine max (mg/dL)		1.23 ± 0.64	1.76 ± 1.02	1.21 ± 0.61	**0.001**
ESR (mm/h)		24.0 [7.0–49.0]	7.0 [5.2–50.2]	25.0 [9.0–49.0]	0.44
CRP (mg/L)		25.2 [5.1–88.6]	12.7 [8.8–94.7]	29.5 [4.5–87.9]	0.82
Sodium (mmol/L)		138.45 ± 3.82	139.00 ± 5.39	138.43 ± 3.75	0.51
Potassium (mmol/L)		4.17 ± 0.57	4.13 ± 0.71	4.17 ± 0.57	0.64
Total cholesterol (mg/dL)		166.0 [141.5–191.5]	181.0 [148.0–222.0]	165.0 [141.0–190.2]	0.15
Triglyceride (mg/dL)		111.0 [79.0–163.0]	105.0 [85.0–125.0]	111.5 [79.0–163.2]	0.29
LDL (mg/dL)		106.0 [84.5–128.0]	128.0 [62.0–146.0]	106.0 [85.0–127.0]	0.52
HDL (mg/dL)		39.0 [33.0–47.0]	43.0 [37.2–52.5]	39.0 [33.0–47.0]	0.09
Angiographic data					
Door to device time (min)		25.0 [19.0–35.0]	30.0 [20.0–45.0]	25.0 [19.0–34.0]	0.21
First medical contact to device time (min)		75.0 [60.0–100.0]	75.0 [60.0–120.0]	75.0 [60.0–100.0]	0.12
Lesion bifurcation		185/1123 (16.5%)	4/56 (7.1%)	181/1067 (17.0%)	0.05
Culprit lesion territory					
SVG		13/1123 (1.2%)	1/56 (1.8%)	12/1067 (1.1%)	0.32
LAD		589/1123 (52.4%)	32/56 (57.1%)	557/1067 (52.2%)	
Lcx		136/1123 (12.1%)	7/56 (12.5%)	129/1067 (12.1%)	
RCA		381/1123 (33.9%)	15/56 (26.8%)	366/1067 (34.3%)	
LM		4/1123 (0.4%)	1/56 (1.8%)	3/1067 (0.3%)	
Pre‐TIMI ≤ 2		875/1123 (77.9%)	51/56 (91.1%)	824/1067 (79.3%)	**0.03**
Post‐TIMI ≤ 2		118/1123 (10.5%)	22/56 (39.3%)	96/1067 (9.2%)	**<0.001**

^a^ Data are presented as mean ± standard deviation or number (%).

^b^ Statistically significant *p* values are bolded.

Abbreviations: CRP, C‐reactive protein; ESR, erythrocyte sedimentation rate; HDL, high‐density lipoprotein; LAD, left anterior descending; Lcx, left circumflex; LDL, low‐density lipoprotein; LM, left main; PCI, percutaneous coronary intervention; RCA, right coronary artery; STEMI, ST‐elevation myocardial infarction; SVG, saphenous vein graft; TIMI, thrombolysis in myocardial infarction.

**Table 2:** Comparison of baseline, echocardiographic, and angiographic characteristics of STEMI patients undergoing primary PCI among follow‐up mortality and alive groups.

Characteristics^a^		Total (*N* = 875)	Follow‐up mortality (*N* = 124)	Alive (*N* = 751)	*p* Value^b^
Demographics					
Age (years)		59.53 ± 12.30	66.50 ± 13.33	58.38 ± 11.74	**<0.001**
Sex					
Male		734 (83.9%)	96 (77.4%)	638 (85.0%)	**0.03**
Female		141 (16.1%)	28 (22.6%)	113 (15.0%)	
Comorbidities					
Hypertension		426/875 (48.7%)	48/124 (38.7%)	378/751 (50.3%)	**0.004**
Diabetes mellitus		280/875 (32.0%)	33/124 (26.6%)	247/751 (32.9%)	0.16
Dyslipidemia		413/875 (47.2%)	33/124 (26.6%)	380/751 (50.6%)	**<0.001**
Smoking		393/875 (44.9%)	34/124 (27.4%)	359/751 (47.8%)	**<0.001**
Coronary revascularization		112/875 (12.8%)	22/124 (17.7%)	90/751 (12.0%)	0.07
Hemodynamic and echocardiographic data					
Left ventricle ejection fraction (%)		42.75 ± 11.34	38.87 ± 13.74	43.25 ± 10.90	**0.004**
Systolic blood pressure (mmHg)		130.05 ± 27.44	119.82 ± 33.75	131.74 ± 25.90	**0.001**
Diastolic blood pressure (mmHg)		74.27 ± 12.05	69.64 ± 15.97	75.03 ± 11.11	**0.001**
Mean arterial pressure (mmHg)		92.86 ± 16.32	86.37 ± 21.03	93.93 ± 15.17	**<0.001**
Pulse pressure (mmHg)		55.78 ± 19.13	50.17 ± 21.97	56.71 ± 18.48	**0.004**
Laboratory data					
White blood cells (×10^9^/L)		11.73 ± 3.67	12.29 ± 4.05	11.64 ± 3.61	0.10
Hemoglobin (g/dL)		14.26 ± 2.02	13.76 ± 2.44	14.34 ± 1.95	**0.02**
Platelets (×10^9^/L)		226.44 ± 70.26	237.81 ± 79.40	224.78 ± 68.73	0.08
Urea (mg/L)		35.78 ± 19.69	48.41 ± 41.92	33.92 ± 12.70	**0.001**
Creatinine (mg/dL)		1.17 ± 0.61	1.42 ± 1.44	1.13 ± 0.33	**0.04**
Creatinine max (mg/dL)		1.24 ± 0.69	1.59 ± 1.56	1.19 ± 0.41	**0.009**
ESR (mm/h)		24.0 [7.0–49.2]	13.5 [6.2–88.2]	25.0 [8.5–46.0]	0.38
CRP (mg/L)		31.0 [5.0–96.2]	20.8 [6.0–108.3]	46.5 [4.8–96.4]	0.71
Sodium (mmol/L)		138.66 ± 3.84	139.06 ± 4.27	138.60 ± 3.77	0.25
Potassium (mmol/L)		4.17 ± 0.58	4.27 ± 0.69	4.16 ± 0.56	0.11
Total cholesterol (mg/dL)		166.5 [141.2–191.0]	173.0 [143.5–207.7]	166.0 [141.0–188.0]	0.35
Triglyceride (mg/dL)		111.0 [80.0–160.2]	104.0 [82.0–170.7]	112.0 [80.0–159.7]	0.29
LDL (mg/dL)		106.0 [85.0–129.5]	106.0 [85.5–142.7]	106.0 [85.0–128.0]	0.65
HDL (mg/dL)		39.0 [34.0–46.0]	41.5 [35.2–49.0]	39.0 [33.0–45.9]	**0.002**
Angiographic data					
Door to device time (min)		25.0 [20.0–35.0]	25.0 [20.0–40.0]	25.0 [19.0–35.0]	0.82
First medical contact to device time (min)		76.0 [60.0–105.0]	75.0 [60.0–110.0]	76.5 [60.0–105.0]	0.15
Lesion bifurcation		136/875 (15.5%)	17/124 (13.7%)	119/751 (15.8%)	0.54
Culprit lesion territory					
SVG		12/875 (1.4%)	1/124 (0.8%)	11/751 (1.5%)	**0.006**
LAD		470/875 (53.7%)	64/124 (51.6%)	406/751 (54.1%)	
Lcx		104/875 (11.9%)	19/124 (15.3%)	85/751 (11.3%)	
RCA		285/875 (32.6%)	37/124 (29.8%)	248/751 (33.0%)	
LM		4/875 (0.5%)	3/124 (2.4%)	1/751 (0.1%)	
Pre‐TIMI ≤ 2		718/875 (82.1%)	106/124 (86.2%)	612/751 (83.6%)	0.47
Post‐TIMI ≤ 2		94/875 (10.7%)	28/124 (22.8%)	66/751 (9.0%)	**≤0.001**

Abbreviations: CRP, C‐reactive protein; ESR, erythrocyte sedimentation rate; HDL, high‐density lipoprotein; LAD, left anterior descending; Lcx, left circumflex; LDL, low‐density lipoprotein; LM, left main; PCI, percutaneous coronary intervention; RCA, right coronary artery; STEMI, ST‐elevation myocardial infarction; SVG, saphenous vein graft; TIMI, thrombolysis in myocardial infarction.

^a^ Data are presented as mean ± standard deviation or number (%).

^b^ Statistically significant *p* values are bolded.

### 3.2 Primary PCI results

The angiographic data demonstrated that the left anterior descending (LAD) coronary artery was identified as the culprit lesion in 589/1123 (52.4%) patients followed by right coronary artery (RCA) and left circumflex (LCX) coronary artery. The first medical contact‐to‐device median time was 75.0 min, and primary PCI was performed with a median door‐to‐device time of 25.0 min in our center. Thrombolysis in myocardial infarction (TIMI) flow grade before angioplasty was significantly different between patients who survived and who demised during admission (*p* value: 0.03). In addition, postprocedural TIMI flow grade 2 and less was significantly higher in patients who died in‐hospital in comparison with survivors (39.3% vs. 9.2%; *p* value: 0.001).

### 3.3 Predictors of in‐hospital mortality

**Figure 2:**
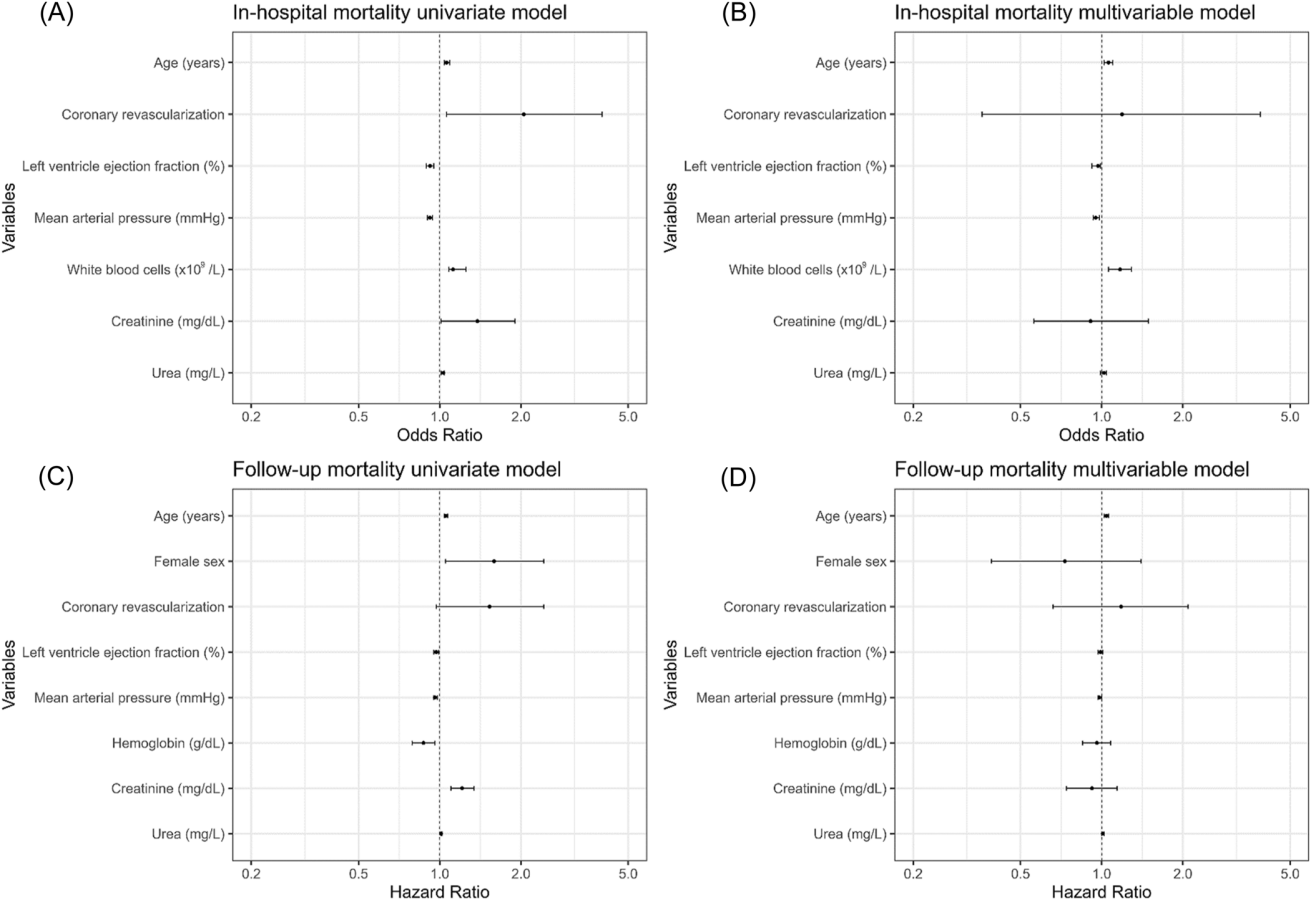
The forest plots present results from regression models. (A) In‐hospital mortality univariate model. (B) In‐hospital mortality multivariable model. (C) Follow‐up mortality univariate model. (D) Follow‐up mortality multivariable mode.

In univariate logistic regression analysis, several predictors of in‐hospital mortality among all STEMI patients undergoing primary PCI emerged. These included advanced age (OR: 1.06; 95% CI: 1.04–1.09; *p* value: <0.001), a history of revascularization (OR: 2.05; 95% CI: 1.06–4.00; *p* value: 0.03), lower LVEF (OR: 0.92; 95% CI: 0.89–0.95; *p* value: <0.001), lower MAP (OR: 0.92; 95% CI: 0.90–0.94; *p* value: <0.001), an increased count of white blood cells (OR: 1.12; 95% CI: 1.08–1.25; *p* value: <0.001), elevated blood/serum creatinine (OR: 1.38; 95% CI: 1.01–1.90; *p* value: 0.04) and blood urea (OR: 1.03; 95% CI: 1.01‐1.04; *p* value: 0.001) upon admission (Figure 2A).

After multivariate logistic regression analysis, the predictors that maintained independent association with in‐hospital mortality were older age (OR: 1.06; 95% CI: 1.02–1.10; *p* value: 0.001), lower LVEF (OR: 0.97; 95% CI: 0.92–0.99; *p* value: 0.02), lower MAP (OR: 0.95; 95% CI: 0.93–0.98; *p* value: 0.002), and an increased count of white blood cells (OR: 1.17; 95% CI: 1.06–1.29; *p* value: 0.002) (Table 3 and Figure 2B).

**Table 3:** Predictors of in‐hospital mortality in STEMI patients undergoing primary PCI.

	Model 1^a^			Model 2^b^		
	Odds ratio	95% CI	*p* Value^c^	Odds ratio	95% CI	*p* Value^c^
Age (years)	1.06	1.04–1.09	**<0.001**	1.06	1.02–1.10	**0.001**
Coronary revascularization	2.05	1.06–4.00	**0.03**	1.19	0.36–3.88	0.76
Left ventricle ejection fraction (%)	0.92	0.89–0.95	**<0.001**	0.97	0.92–0.99	**0.02**
Mean arterial pressure (mmHg)	0.92	0.90–0.94	**<0.001**	0.95	0.93–0.98	**0.002**
White blood cells (×10^9^/L)	1.12	1.08–1.25	**<0.001**	1.17	1.06–1.29	**0.002**
Creatinine (mg/dL)	1.38	1.01–1.90	**0.04**	0.91	0.56–1.49	0.72
Urea (mg/L)	1.03	1.01–1.04	**<0.001**	1.02	0.99–1.04	0.15

Abbreviations: CI, confidence interval; PCI, percutaneous coronary intervention; STEMI, ST‐elevation myocardial infarction.

^a^ Univariate binary logistic regression model.

^b^ Multivariate binary logistic regression model adjusted for age, coronary revascularization, left ventricle ejection fraction, mean arterial pressure, white blood cells, creatinine, and urea.

^c^ Statistically significant *p* values are bolded.

### 3.4 Predictors of long‐term mortality

**Figure 3:**
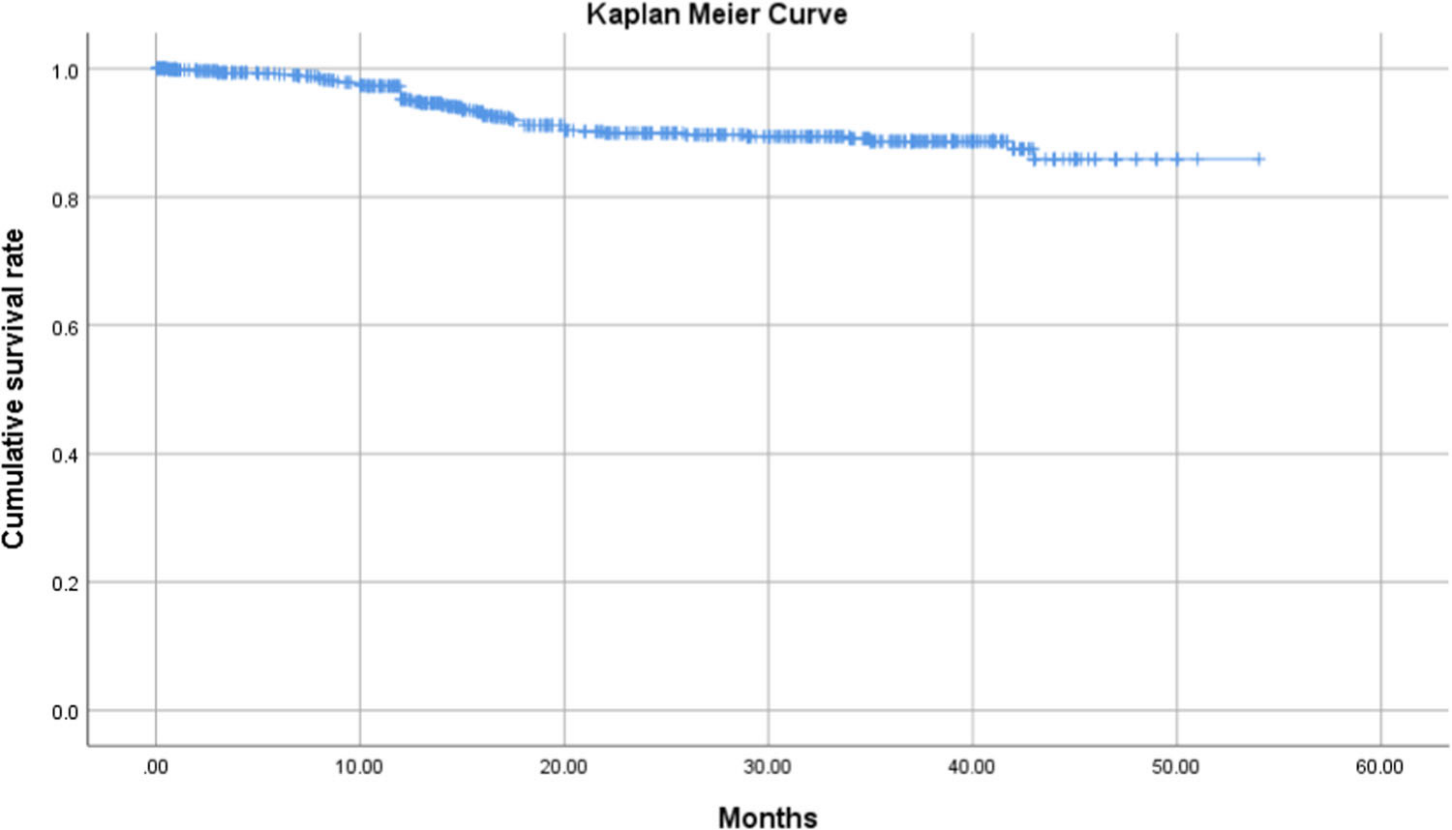
The Kaplan–Meier curve presents the median follow‐up time for the study population.

A total number of 875 patients were followed over a median duration of 21.8 months (Figure 3). Following univariate Cox regression analysis, predictors of long‐term mortality among all STEMI patients undergoing primary PCI were identified as advanced age (hazard ratio [HR]: 1.05; 95% CI: 1.04–1.07; *p* value: 0.001), female sex (HR: 1.59; 95% CI: 1.05–2.43; *p* value: 0.03), a history of revascularization (HR: 1.53; 95% CI: 0.97–2.43; *p* value: 0.07), lower LVEF (HR: 0.97; 95% CI: 0.95–0.99; *p* value: 0.001), lower MAP (HR: 0.96; 95% CI: 0.95–0.98; *p* value: 0.001), lower hemoglobin (HR: 0.87; 95% CI: 0.79–0.96; *p* value: 0.004), higher blood/serum creatinine (HR: 1.21; 95% CI: 1.10–1.34; *p* value: 0.001), and higher blood urea (HR: 1.01; 95% CI: 1.01–1.02; *p* value: 0.001) upon admission (Figure 2C).

Upon performing multivariate Cox regression analysis, older age (HR: 1.04; 95% CI: 1.02–1.06; *p* value: <0.001), lower MAP (HR: 0.98; 95% CI: 0.97–1.00; *p* value: 0.04), and higher blood urea (HR: 1.01; 95% CI: 1.00–1.02; *p* value: 0.004) were demonstrated to be independently associated with long‐term mortality during the follow‐up period in STEMI patients undergoing primary PCI (Table 4 and Figure 2D).

**Table 4:** Predictors of long‐term mortality in STEMI patients undergoing primary PCI.

	Model 1^a^			Model 2^b^		
	Hazard ratio	95% CI	*p* Value^c^	Hazard ratio	95% CI	*p* Value^c^
Age (years)	1.05	1.04–1.07	**0.001**	1.04	1.02–1.06	**<0.001**
Female sex	1.59	1.05–2.43	**0.03**	0.73	0.39–1.40	0.35
Coronary revascularization	1.53	0.97–2.43	0.07	1.18	0.66–2.09	0.58
Left ventricle ejection fraction (%)	0.97	0.95–0.99	**0.001**	0.99	0.97–1.01	0.22
Mean arterial pressure (mmHg)	0.96	0.95–0.98	**0.001**	0.98	0.97–1.00	**0.04**
Hemoglobin (g/dL)	0.87	0.79–0.96	**0.004**	0.96	0.85–1.08	0.47
Creatinine (mg/dL)	1.21	1.10–1.34	**0.001**	0.92	0.74–1.14	0.43
Urea (mg/L)	1.01	1.01–1.02	**0.001**	1.01	1.00–1.02	**0.004**

Abbreviations: CI, confidence interval; PCI, percutaneous coronary intervention; STEMI, ST‐elevation myocardial infarction.

^a^ Univariate Cox regression model.

^b^ Multivariate Cox regression model adjusted for age, sex, coronary revascularization, left ventricle ejection fraction, mean arterial pressure, hemoglobin, creatinine, and urea.

^c^ Statistically significant *p* values are bolded.

## 4 DISCUSSION

Primary PCI has been recommended as the treatment of choice in STEMI patients. However, even with an increase in PCI‐capable centers, the emergence of new generation stents, and the continual refinement of guidelines, a significant mortality rate remains among patients who undergo primary PCI in a timely manner. This study has helped identify predictors of two main outcomes: in‐hospital mortality and long‐term mortality post‐discharge. These predictors include older age, lower LVEF upon admission, reduced MAP, and elevated counts of white blood cells. For long‐term mortality, risk factors also include older age, decreased MAP, and heightened blood urea level.

In this study, age was revealed to be related to both in‐hospital (OR: 1.06) and long‐term mortality (HR: 1.04) after primary PCI. Prior investigations indicated the effect of older age on the outcome of patients who underwent primary PCI in such a way that patients aged 75 years or older had a higher likelihood of experiencing complications, leading to in‐hospital or long‐term mortality.^21^ These data are consistent with our results. However, numerous studies suggested that women are more likely than men to develop side effects after PCI, regardless of age, and young women have a higher mortality risk after an elective PCI.^22^, ^23^, ^24^ Although sex differences were significant in univariate analysis, it was not an independent risk predictor for long‐term mortality.

Interestingly, a history of smoking was related to both in‐hospital and long‐term mortality, yet the correlation was in favor of a protective effect (Tables 1 and 2). According to recent literature, the “smoker's paradox,” states that smokers may have improved survival rates after AMI. This phenomenon occurs because smokers experience MI at a much younger age than nonsmokers do. However, it continued to be noticeable that the smoker's paradox occurs even after adjustment for age differences between smoking groups.^25^ This paradox was mainly observed during the thrombolytic era. However, there is little and incoherent support for the smoker's paradox in the current era of PCI therapy. Due to these effects, we did not enter this variable into further analysis.^26^

In this research, MAP was found to be associated with both in‐hospital (OR: 0.95) and long‐term mortality (HR: 0.98) following primary PCI. Previous studies have highlighted a significant role of hemodynamic factors on mortality in patients with STEMI receiving thrombolytic therapy.^27^, ^28^, ^29^ The TIME‐II trial identified a prognostic role for HR > 100, SBP < 100 mmHg, and Killip class to predict 30‐day mortality after STEMI.^30^, ^31^ Moreover, in a sub‐study of the Assessment of Pexelizumab in Acute Myocardial Infarction (APEX‐AMI) trial, it was shown that SBP (HR 0.86/10 mmHg increments, 95% CI: 0.82–0.90), heart rate (HR 1.45/10 beat increments, 95% CI: 1.31–1.59) and Killip class of ≥3 (HR 4.24, 95% CI: 2.97–6.08) are significant predictors of 90‐day mortality in patients with STEMI who undergo primary PCI within 6 h of pain onset.^32^ Recent studies have suggested a high rate of mortality and rapid deterioration among STEMI patients who present with cardiogenic shock.^33^ In this study, 3.7% of patients presented with shock upon admission. However, the lack of data on pulmonary capillary wedge pressure and cardiac index prevented us from definitively diagnosing cardiogenic shock. Therefore, these studies indicate that hemodynamic parameters at presentation can significantly predict short‐term, in‐hospital, and long‐term mortality among patients undergoing primary PCI for STEMI. These findings emphasize the importance of meticulous monitoring of patients with hemodynamic compromise upon presentation to initiate hemodynamic support as needed.

In addition, we have observed a correlation between LVEF and in‐hospital mortality (OR: 0.97). This finding is consistent with current literature where left ventricular dysfunction remains a significant predictor of both early and late mortality.^34^ Whenever possible, it should be advised to assess left ventricular function before PCI because it is crucial for accurate risk stratification and optimal patient management.^35^

In our study, we found blood urea to be associated with follow‐up mortality (HR: 1.01). The relationship between blood urea nitrogen and mortality in patients with coronary artery disease has not been extensively studied though recent studies are beginning to highlight the importance of blood urea in CVDs. The best predictor of in‐hospital mortality in patients with acute heart failure is the elevated blood urea level at admission time according to several studies.^36^, ^37^ Blood urea is also a central biomarker in seriously ill patients who do not have acute heart failure.^38^ It may show hemodynamic deterioration as well as neurohormonal activation of the sympathetic nervous system, renin‐angiotensin‐aldosterone system, and secretion of arginine vasopressin.^39^ Despite the longstanding recognition of renal insufficiency as a risk factor for CVDs, particularly coronary artery disease, in our study, blood/serum creatinine did not emerge as a significant predictor. Multiple mechanisms, including increased systemic inflammation,^40^ dyslipidemia,^41^ and endothelial dysfunction^42^ are responsible for the increased rate of adverse outcomes in patients with impaired renal function. A recent study by Jia et al. in patients with STEMI undergoing primary PCI demonstrated that after adjusting for confounding factors, the risk of in‐hospital and all‐cause death was approximately four times higher in patients with a GFR of 30–45 mL/min/1.73 m^2^ (OR: 4.15; 95% CI: 1.44–11.96) and eight times higher when GFR was <30 mL/min/1.73 m^2^ (OR: 8.15; 95% CI: 2.45–27.04).^43^ Similar results were found by Sabroe et al. showing a higher risk of 30‐day mortality after primary PCI for patients with STEMI presenting with a GFR of 30–60 mL/min/1.73 m^2^ (HR: 2.71; 95% CI: 2.09–3.51) and a GFR of <30 mL/min/1.73 m^2^ (HR: 7.09; 95 CI: 4.82–10.44) indicating a pronounced adverse effect in patients with severe renal dysfunction at presentation.^44^ According to these findings, renal function plays a major role in the prognosis of patients with STEMI undergoing primary PCI, and improving renal function by controlling blood urea rather than blood/serum creatinine through preventive strategies such as adequate serum therapy and minimizing administration of contrast media could potentially reduce the risk of mortality.

TIMI flow grade 3, long known as an indicator of successful PCI, denotes sufficient coronary blood flow and revascularization of the infarct‐related artery. Previous studies have suggested poor short‐term and follow‐up clinical outcomes in patients with STEMI and a postprocedural TIMI flow grade ≤2.^45^, ^46^ In a study by Mehta et al. on patients with STEMI complicated by cardiogenic shock, it was shown that in‐hospital mortality was nearly twice as high in patients with postprocedural TIMI flow grade 2 compared to patients with TIMI flow grade of 3.^47^ Similarly, another study assessing STEMI patients with cardiogenic shock showed that a TIMI flow ≤2 post‐PCI is a significant predictor of in‐hospital mortality (OR: 2.57).^48^ In this study, we found that a final TIMI flow grade of 2 or less also significantly increases the risk of in‐hospital and total mortality in an unselected group of STEMI patients undergoing primary PCI (Tables 1 and 2). However, cardiogenic shock appears to be a predictor of poor final TIMI flow grade, underscoring the need for special attention in this subgroup of patients.^49^, ^50^

Our study demonstrated a significant correlation between the total count of white blood cells and in‐hospital mortality (OR: 1.17). This finding is considered less in guidelines which primarily highlight other prominent indicators such as multivessel disease, renal dysfunction, anemia, SBP, B‐type natriuretic peptide, and troponin. Yet, the total white blood cells can be utilized as a comprehensive marker to predict future adverse events following any treatment for AMI and is extremely important for patient risk stratification.^51^

## 5 STRENGTHS AND LIMITATIONS

This study has several notable strengths that contribute to its reliability and comprehensive analysis. First, the study was designed with a large sample size, ensuring sufficient statistical power to draw meaningful interpretations from the results. In addition, the inclusion of long‐term follow‐up for some patients, exceeding 1 year, enables a thorough assessment of risk stratification for both in‐hospital and long‐term mortality. Furthermore, the study incorporates a wide range of parameters including demographic, laboratory, and angiographic data. This comprehensive approach allows for the consideration of various potential risk factors, ensuring a more thorough evaluation of the subject under investigation.

There are several limitations to this study. First, this was a single‐center study which may limit the generalizability of the results. Second, this study was limited by its retrospective nature which is subject to bias. Third, it is important to note that the medication history of the patients was collected through self‐report, which introduces a potential recall bias. Consequently, due to this potential bias, we were unable to incorporate these data into our assessment.

## 6 CONCLUSION

This study showed that factors such as age, LVEF, MAP, and white blood cell count have significant prognostic roles in determining in‐hospital mortality of STEMI patients undergoing primary PCI within 12 h of pain onset. Furthermore, age, MAP, and blood urea have independent roles in determining long‐term mortality.

## AUTHOR CONTRIBUTIONS

All authors have read and approved the final version of the manuscript. The corresponding author had full access to all of the data in this study and took complete responsibility for the integrity of the data and the accuracy of the data analysis.

## CONFLICT OF INTEREST STATEMENT

The authors declare no conflict of interest.

## ETHICS STATEMENT

The protocol of this study followed the declaration of Helsinki and was approved by the ethics committee of the Tehran University of Medical Sciences (IR.TUMS.MEDICINE.REC.1398.244). All participants in the study gave written informed consent before their inclusion.

## TRANSPARENCY STATEMENT

The lead author Shahrokh Karbalai Saleh affirms that this manuscript is an honest, accurate, and transparent account of the study being reported; that no important aspects of the study have been omitted; and that any discrepancies from the study as planned (and, if relevant, registered) have been explained.

## Data Availability

Shahrokh Karbalai Saleh, as the corresponding author of the study, will make the data supporting the findings available upon reasonable request. He has complete access to all the data in the study and assumes full responsibility for the data's integrity and the accuracy of the data analysis.
